# Nurses’ perceptions, experience and knowledge regarding artificial intelligence: results from a cross-sectional online survey in Germany

**DOI:** 10.1186/s12912-024-01884-2

**Published:** 2024-03-27

**Authors:** Domenic Sommer, Lukas Schmidbauer, Florian Wahl

**Affiliations:** Deggendorf Institute of Technology, Dieter-Görlitz-Platz 1, Deggendorf, 94469 Bavaria Germany

**Keywords:** Nurse, Artificial intelligence, Healthcare, Nursing education, Germany

## Abstract

**Background:**

Nursing faces increasing pressure due to changing demographics and a shortage of skilled workers. Artificial intelligence (AI) offers an opportunity to relieve nurses and reduce pressure. The perception of AI by nurses is crucial for successful implementation. Due to a limited research state, our study aims to investigate nurses’ knowledge and perceptions of AI.

**Methods:**

In June 2023, we conducted a cross-sectional online survey of nurses in Bavaria, Germany. A convenience sample via care facilities was used for the questionnaire oriented on existing AI surveys. Data analysis was performed descriptively, and we used a template analysis to evaluate free-text answers.

**Results:**

114 (♀67.5 %, ♂32.5 %) nurses participated. Results show that knowledge about AI is limited, as only 25.2 % can be described as AI experts. German nurses strongly associate AI with (i) computers and hardware, (ii) programming-based software, (iii) a database tool, (iv) learning, and (v) making decisions. Two-thirds of nurses report AI as an opportunity. Concerns arise as AI is seen as uncontrollable or threat. Administration staff are seen as the biggest profiteers.

**Conclusion:**

Even though there is a lack of clear understanding of AI technology among nurses, the majority recognizes the benefits that AI can bring in terms of relief or support. We suggest that nurses should be better prepared for AI in the future, e.g., through training and continuing education measures. Nurses are the working group that uses AI and are crucial for implementing nursing AI.

**Supplementary Information:**

The online version contains supplementary material available at 10.1186/s12912-024-01884-2.

## Background

The rise of artificial intelligence (AI), propelled by advancements like ChatGPT, has heightened discourse around its varied uses, benefits, and challenges [1]. Originating in the 1950s, AI has evolved with technology, attracting increasing interest [2–4].

### Ontology and terminology

To understand the nuances of AI and its subfields, it’s crucial to delineate the terms AI, machine learning (ML), and deep learning (DL). The definition of these different terms is often lacking in current literature, although this separation is crucial according to Castagno et al. [5] and Graziani et al. [3].

- AI is about machines that perform pure calculations. They are not based on logical thinking and merely imitate human characteristics [6].
- ML and DL, subsets of AI, encompass data-driven learning and adaptation, enhancing predictions and decisions autonomously [4, 7].

AI, the term we focus on, symbolizes a new frontier in human-like intelligence. AI amalgamates various cutting-edge technologies, including ML, Computer Vision, and Natural Language Processing. Sheikh et al. [7] describe AI as a relatively recent technology, often perceived as a black box, designed to execute tasks requiring human intellect, including speech comprehension, pattern recognition, and decision-making. The World Economic Forum defined AI as an ‘act by sensing, interpreting data, learning, reasoning, and recommending’ [8]. However, this taxonomy appears intricate and multifaceted, encompassing numerous social, ethical, legal, and technical aspects, without yielding an universal definition [3].

In our study, we simplify^1^ AI as an intelligent system that analyses its surroundings and autonomously acts, to fulfill specific objectives [7].

### Relevance and appliance in nursing context

European healthcare, challenged by an aging population and nursing staff shortages, faces increasing demands and work-related stress [9, 10]. Consequently, AI-supported solutions are emerging as key responses to these pressures [8, 10]. Economically, AI in healthcare is significant, with spending projected to reach 36.1 billion USD by 2025 [4].

AI applications, ranging from conversational agents in customer service to analytical tools in image and video analysis, are transforming various sectors, including healthcare, and facilitating smart hospitals [11]. This transformation is met with mixed public reactions, from optimism to concerns over depersonalization and job displacement in healthcare [12]. The rapid expansion of AI in healthcare promises significant advancements, notably in predictive analysis and virtual assistance, reshaping patient care and nursing practices in the near future [5, 13]. AI’s integration into healthcare is poised to alleviate workforce strains by supporting nursing staff in areas like documentation, workflow optimization, and decision-making, enhancing care quality [10, 14]. The goal in developing AI applications is to support nurses in their workflow. In this way, the role of the nurses, which is essentially human care, can be retained [1, 15].

### Research about nurses perceptions

Nurses, numbering 27 million globally, are the largest group in healthcare and play a critical role in evaluating and integrating AI technologies due to their insights and interfaces with various professional groups [16, 17]. Nurses central position in patient care and implementation of AI in clinical settings is key to the successful adoption of AI technologies [5, 13, 18]. The rise of AI in healthcare heightens the need for human-computer interaction (HCI) research and nurses involvement in AI development [17]. However, the specifics of nurses involvement are yet to be defined [1, 17, 19, 20].

The current international research on nursing AI is multidimensional, focusing on technical aspects [17, 21] and envolved on perceptions of AI, as shown in Table 1.

**Table 1:** Related work about nurses’ perceptions on AI

AI Categories	Subitems	Sources
Knowledge	Uncertainties in terminology and lack of AI knowledge	[5, 17, 22]
	Lack of experience & application in nursing	[5, 17, 19, 23, 24]
Attitudes	AI enhances nursing outcomes & reliefs staff^a^	[5, 13, 15, 25, 26]
	AI increases efficiency & reduces costs^a^	[15, 26, 27]
	AI is available, user-friendly & easy to use^a^	[27, 28]
	AI changes organizations & workflows^b^	[5, 13, 15]
	AI changes leadership^b^	[15, 29]
	AI influences nursing roles^b^	[5, 13, 15]
	AI isn’t capable enough to replace human interaction^c^	[10, 15]
	AI as frightening threat & mistrust^c^	[5, 8, 25, 26]
	Unawareness of advantages & applications^c^	[8, 29–31]
	Worries about patient relationship & safety^c^	[10, 13, 27]
Barriers	Anxiety of job loss and full automation	[25, 27, 30]
	Missing education and sensitization	[5, 22, 25]
	Lack of data and interfaces to train AI	[8, 23, 27]
	Errors, unexpected results and AI trustworthiness	[20, 25]
	Regulatory Frameworks and Data Protection	[5, 21, 23, 27]
Facilitators	Positive Outcomes increases intention to use AI	[1, 25]
	Proactive define AI & advocate for patients	[13, 21]
	Empathetic & personalised AI applications	[12, 27]
	Application in health monitoring, documentation, communication, & clinical decisions support	[20, 23, 24]
	Training & Information about AI	[20, 30, 31]
	IT clinicians & technical infrastructure	[8, 15, 20]
Further Research	AI applications & outcomes, esp. relief of nurses	[1, 4, 10, 15, 17, 21, 23]
	Nursing perspective, acceptance, nursing role	[12, 13, 18, 20, 27, 29]
	Acceptance & user-centered design	[31, 32]
	Ethical, social & legal implications	[8, 17, 22, 23, 25]
	Limitations: response, study sample & lab settings	[1, 5, 15, 17, 23, 27]

^a^positive picture ^b^neutral picture ^c^negative picture

Most studies indicate a limited understanding and experience with AI among nurses [5, 17, 22, 23]. Castagno et al. 2020 [5] found that 64 % of nurses in Britain had no previous contact with AI, and 87 % didn’t know the difference between AI terms. In Germany, two-thirds of the public don’t understand AI [22]. Even though there is a knowledge gap, a sense of optimism toward AI is evident. Over 70 % of nurses agree that AI can catalyze transformation in nursing through better health promotion, personalized treatments, and automation in administration as well as routine tasks [26]. Nurses positively state that AI enhances health outcomes, relieves staff, and reduces costs [5, 13, 15, 25–27]. Chew et al. [27] found out, that healthcare workers perceive low-threshold access to AI. Although various AI benefits, nurses can’t estimate how their roles will be affected [13]. In addition, nurses think, that AI isn’t capable enough, because nursing is seen as human-interaction driven, holistic, and adoptable [10, 15].

Contrary to neutral and positive assumptions, 40% of 98 healthcare professionals see AI as ‘potentially more dangerous than nuclear weapons’ [5]. Concerns include unawareness, mistrust, anxiety, worries about the patient relationship and job replacement [5, 8, 10, 13, 25–27]. Despite AI’s benefits, the practical implementation encounters barriers [29]. Integrating AI is hindered by fears, missing sensitization, and a lack of data [8, 22, 23, 25, 27, 30]. In addition, errors are problematic in AI usage, and data protection regulations stop nurses from using AI [20, 23, 27]. Moreover, positive outcomes, empathetic and personalized solutions, IT clinicians that support the process, AI education, and active involvement of nurses in AI development facilitate AI in healthcare [1, 8, 12, 13, 20, 27]. Nurses notice applications for health monitoring, documentation, communication, and decision support to foster AI [20, 23, 24].

Current research reveals limited AI knowledge and varied attitudes among nurses. Studies on nurses’ perceptions of AI face challenges like small samples and sampling bias [15, 17, 21, 27]. The need for updated research is underscored by rapid technological changes and healthcare setting variances [1, 4, 10, 18]. With most international research centered on the US, the applicability to German healthcare is uncertain [18]. Hence, our study focuses on understanding AI perception in Germany’s unique healthcare context, aiming to guide user-oriented AI development in nursing.

## Methods

Between June 5^th^ and June 30^th^, 2023, we conducted an online survey regarding nurses’ knowledge, perception, and experience of AI in Germany, Bavaria. Nurses of all ages and care settings were included, mainly from geriatric, inpatient, and clinical nursing.

### Objective and research question

Our study seeks to enhance the understanding of nurses’ perceptions of AI in Germany, where AI adoption is emerging. We aim to gather empirical insights to guide user-centric AI development in nursing. Additionally, we aim to identify potential anxieties or misconceptions to inform practical educational initiatives and provide an overview of the current status of AI perception in nursing for policymakers, technicians and nursing managers. The research questions (RQs) we investigate are:

1. To what extent are nurses in Germany informed about AI, and what attitudes do they hold towards using AI in nursing?
2. Which areas of AI application are most recognized by nurses, and which ones do they perceive as most promising in nursing?
3. Based on nurses’ self-assessment, which healthcare professions would reap the greatest benefits from AI integration, and why?

### Study instrument

Our questionnaire development was informed by a narrative literature review, sourcing relevant literature from databases like PubMed, CINAHL, Medline, and Web of Science, supplemented by Google Scholar. Key terms related to nurses’ perceptions, experience, and knowledge of AI guided the search. The most influential study for our questionnaire design was the BIDT study [22], allowing later comparison with broader population data. While models like the Technology Acceptance Model (TAM) and the Unified Theory of Acceptance and Use of Technology (UTAUT) were considered, their applicability seemed limited due to their focus on real-life technology use [33]. Instead, we adapted models tailored for nursing, as described by Gaughan et al. [34]. The final survey, shaped by existing studies [5, 22, 25, 30], integrated items from the German BIDT study [22] and Swan et al. [26] for comparability. A pre-test involving 6 nurses ensured the survey’s clarity and reliability, leading to minor adjustments in wording.

The final survey, provided in the Appendix, comprises twelve questions categorized into three sections: (i) demographic data (age group, gender, working field, education), (ii) knowledge about AI, and (iii) perceptions of AI. The survey primarily uses nominal and ordinal scales for quantitative (close-ended) data. To enrich our data with qualitative insights, we included open-ended questions. Participants were prompted to describe in their own words what they understood by AI. At the end of the survey, participants were invited to offer comments or feedback on AI in a free-text format. This open-ended approach provided diverse, nuanced perspectives on AI, revealing varying levels of understanding and attitudes among nurses. The combination of quantitative and qualitative data from these questions effectively addressed the research questions, offering a balanced view of nurses’ knowledge and perception of AI.

### Data collection

Data for our cross-sectional study was collected via a self-developed online survey, using the tool Lime-Survey. The questionnaire was distributed in a convenience sample through emails and multipliers to nursing facilities in Lower Bavaria and Upper Palatinate. Participants consented voluntarily and anonymously, with the study adhering to ethical guidelines. Study participants opt-in voluntarily for their consent after study information. In all cases, completing the survey took under five minutes. Questions about nurses’ prior experiences with AI in their work were based on dimensions resulting from a scoping review [35]. The survey design and its distribution aimed to capture a broad and representative sample of nurses’ perspectives on AI, despite limitations like sampling bias and non-validated items, discussed under Discussion section.

### Data analysis

Our reporting of results adheres to our survey structure and is informed by methodologies used in the BIDT Study [22]. Quantitative data analysis was performed using IBM SPSS (Version 29). We analyzed demographic data (questions A.2.1-A.2.4) including age, gender, facility type, and education level, along with AI knowledge (A.3.1, A.3.3) and perception (A.4.1, A.4.2) responses, using frequencies and percentages.

We utilized bivariate statistics, including Spearman’s rank correlation and Cramer’s V, to analyze the impact of demographic factors on self-rated AI understanding and knowledge. These methods helped identify key patterns and associations between demographic variables and AI knowledge/perception. However, due to our study’s limited sample size, these results should be considered indicative, leading to the need of further research [36]. The significance levels were set at $\alpha = 0.05$ for significance and $\alpha = 0.01$ for high significance, aiming for a 95% confidence in minimizing the $\beta$-error. Cross-tabulation and chi-square tests were also applied to assess differences and relationships among demographic and AI-related variables.

In addition to the analysis of close-ended data, we employed a qualitative template analysis for the open-ended data to create joint displays. Therefore, questions A.3.2 and A.4.3 were free text fields that were analyzed with a structured qualitative template analysis according to Mayring [37] to determine categories for answering our RQ. The units of analysis were the free text fields of the questionnaire. To determine the category system, we chose a deductive approach by orienting ourselves on already known possible categories based on the literature research. In the next step, the text modules were searched for references and a coding of the relevant passages was carried out. The references were edited and extracted. Based on the coding, the original category system was adapted and a second run was started. Table 2 gives an overview of the final category system. The analysis revealed four main categories. These are (i) Knowledge and definition of AI, (ii) Opportunities and perceived benefits, (iii) Anxiety and disadvantages, and (iv) Facilitators and future needs. The analysis identified several subcategories for each category as shown in Table 2.

**Table 2:** Categories of the qualitative template analysis

Categories	Subcategories	Description for Items
Knowledge and definition of AI	Computer-based	AI as a hardware-based solution
	Programming-based	AI as a (text-generating) program
	Input-/information-/data-based	AI as system relying on data
	Learning	AI as a developing thing
	Decision-making	AI as a decision helper
	Human-like	AI as a natural, human-like interaction
	Robot-like	AI as a machine, a robot
	Naming AI providers and tools	AI as e.g. ChatGPT or Mindjourney
Opportunities and perceived benefits	Support and relief in patient care	AI as helper for patient care tasks
	Knowledge acquisition	AI as a tool to find information
	Helping in administrative tasks	AI as writing/ documentation support
	Assistance systems	AI with robots as assistance
Anxiety and disadvantages	Fears of job replacement	AI as threat for nursing jobs
	Impersonally	AI as a threat to social relationships
	Mistakes	AI as error source due to incomplete data
	Insufficient Outcome	AI as not comprehensive to human output
	Costs and implementation efforts	AI as expensive in implementing
Facilitators and future needs	Sensitization	AI information is necessary for nurses
	Refinancing	AI usage in health facilities needs financing
	Data	AI usage in health facilities needs data
	User-friendliness	AI should be easy to use and simple
	Use cases	AI should be helpful and practicable

## Results

The results are presented following the survey structure, including (i) demographics, (ii) an understanding of AI, (iii) use cases, (iv) threats, and (v) benefits.

### Demographic data

A total of 114 nurses completed our survey. As Table 3 shows, two-thirds of our participants were female, and most nurses (29.2 %) were 31 to 40 years old. Regarding the form of the facilities, 39.8 % of nurses work in inpatient long-term care, followed by hospitals (18.6 %) and outpatient care (15.9 %). One-quarter work in other care settings, like specialized care. Moreover, our study population has high education levels, like university degrees (31.0 %) and completed apprenticeships (29.2 %).

**Table 3:** General characteristics of study participants (self-intended, N=113 except gender)

Variable	Category	n ( %)
Gender	Female	77 (67.5 %)
	Male	37 (32.5 %)
	Diverse	0 (00.0 %)
Age group	under 21 years	1 (00.9 %)
	21-30 years	23 (20.4 %)
	31-40 years	33 (29.2 %)
	41-50 years	24 (21.2 %)
	51-60 years	26 (23.0 %)
	over 60 years	6 (05.3 %)
Facility form	Inpatient long-term care	45 (39,8 %)
	Outpatient care	18 (15,9 %)
	Nursing (hospital/clinic)	21 (18.6 %)
	Other	29 (25,7 %)
Education level	Secondary school diploma	21 (18.6 %)
	Higher School or specialized secondary school	16 (14.2 %)
	Completed vocational training	33 (29.2 %)
	University degree (at least Bachelor’s degree)	35 (31.0 %)
	Other	6 (05.3 %)

### Understanding of AI

Table 4 shows nurses’ self-assessment of AI knowledge, measured by five items.

**Table 4:** Knowledge of nurses about AI (self-intended, survey order, N=113)

Variable	Category	n ( %)
How much do you know about AI?	I would describe myself as an AI expert.^a^	2 (01.8 %)
	I can explain well what is meant by it.^a^	26 (23.4 %)
	I know roughly what is meant by it.^b^	67 (60.4 %)
	I know the term, but I don’t know what it means.^b^	14 (12.6 %)
	I have not heard the term before.^b^	2 (01.8 %)

^a^Summarized as ‘AI Connoisseurs/ Experts’ (25.2%), ^b^ ‘AI Non-Connoisseurs/-Experts’ (74.8%)

According to Stürz et al. [22], nurses who describe themself as an AI expert and nurses who can explain well what AI means can be summarized as ‘AI Connoisseurs’. Using this simplification, 25.2 % nurses state to have substantial AI knowledge, are ‘AI Connoisseurs’. Conversely, 74.8 % nurses, in summary, lack a solid AI understanding. Gender differences in self-reported AI knowledge were observed: 42.9 % (15 out of 35) of men and 17.3 % (13 out of 75) of women identified themselves as ‘AI Connoisseurs’.

To examine the association between AI knowledge and gender, $\chi ^2$-tests ($\chi ^2$) were conducted. The results indicated a significant association between gender and AI knowledge ($\chi ^2$ value = 12.363, *p* = 0.015), with a Cramer’s V correlation of 0.335. Additionally, the type of healthcare facility ($\chi ^2$ value = 22.078, *p* = 0.037, Cramer’s V = 0.257) and educational level ($\chi ^2$ value = 46.063, *p* = 0,001, Spearman’s correlation = 0.279) also showed significant influences on AI knowledge. At the same time, age was not a significant factor. Due to low cell counts in our tests, these results should be interpreted cautiously, indicating the need for further research with larger samples.

In addition, study participants’ definitions of AI offer a multidimensional understanding that extends beyond the quantitative assumptions, as emphasized in the following quotes. AI is seen as a dynamic tool with the potential to learn and expand its capabilities (Text 1, para 55). The qualitative analysis reveals that nurses associate AI with (i) computers and hardware, (ii) software, (iii) human-like characteristics exemplified by natural language processing, (iv) a data-driven tool capable of (v) learning, and (vi) decision-making. We recognized a dichotomy in nurses’ perspectives, describing AI with human-like attributes and as a more mechanical, machine-like tool. Participants often see AI as a tool that simulates human interactions or replicates human-like behaviour. This view is further influenced by AI’s association with robots and machines, recognizing that AI is frequently integrated as software into robotic systems. Nurses also name AI providers, such as OpenAI (Text 1, para. 48).

*‘AI is a machine with practices to perform and learn from data’* (Text 1, para. 34)

*‘AI is programmed by humans and is intended to be used in multiple domains to make decisions, perform actions, and perform everyday human tasks within its programming and to identify and solve problems.’* (Text 1, para. 36)

*‘AI means using robots which can react to movements or speech.’ *(Text 1, para. 83)

*‘AI [...] can act similarly to a human, e.g., write text, act human, etc.’* (Text 1, para. 31)

*‘AI is a machine’s ability to imitate human abilities.’* (Text 1, para. 72)

*‘AI can decide and learn alone, considering markers taught to it.’* (Text 1, para. 15)

Furthermore, nurses recognize that AI is not a ready-to-use solution but requires data processing and preparation (Text 1, para. 11). Nurses understand the critical role of data quality in shaping AI as a decision-making instrument (Text 1, para. 55).

### Use-cases and applications

For the fields of application of AI in nursing (Table 5), nurses are most familiar with patient monitoring (55.7 %) and route planning (47.7 %) as well as AI-aided nursing documentation (43.7 %). Nurses know least about the AI areas of care prediction (38.6 %), nursing diagnosis (31.8 %), and wound management (21.6 %).

**Table 5:** Application fields of AI in nursing (self-intended, descending order, N=88)

Variable	Category	n ( %)
Which application areas^a^ of AI in nursing do you know?	Patient monitoring (e.g. vital signs, sleep)	49 (55.7 %)
	Route planning	42 (47.7 %)
	Nursing documentation	38 (43.2 %)
	Patient care prediction (e.g. fall detection)	34 (38.6 %)
	Making nursing diagnoses	28 (31.8 %)
	Wound management	19 (21.6 %)

^a^According to Seibert et al. [23] all displayed AI applications exist and are beneficial for nursing

Our qualitative data reveals AI’s utility extends to patient monitoring, routine nursing tasks, assessing ‘drug compatibility effects and interactions’ (Text 2, para. 124), enhancing ‘patient care’ (Text 2, para. 127), and assisting with ‘intelligent incontinence care’ (Text 2, para. 130). Additionally, nurses recognize AI’s supportive role in administrative functions, such as assistance in ‘staff planning’ (Text 2, para. 125) and ‘service scheduling’ (Text 2, para. 129). Furthermore, AI has potential in ‘housekeeping activities’ (Text 2, para. 134) through AI-based cleaning robots. AI-supported technical assistance, like active exoskeletons, were also highlighted (Text 2, para. 128).

### Opportunities and positive AI perception

Table 6 displays that nearly two-thirds (65.7 %) view AI positively as an opportunity for nursing, while a smaller fraction (13.7 %) perceives AI negatively as a threat or danger. Additionally, 21.6 % of respondents could not categorize AI either way, indicating that they could not judge due to a lack of knowledge.

**Table 6:** Perception of nurses about AI (self-intended, N=103)

Variable	Category	n ( %)
Do you see AI in nursing more as an opportunity or more as a threat?	Exclusively as an opportunity^a^	14 (13.7 %)
	Rather as an opportunity^a^	53 (52.0 %)
	Rather as a danger^b^	13 (12.7 %)
	Exclusively as a danger^b^	1 (01.0 %)
	I do not know, I cannot judge	22 (21.6 %)

^a^Summarized as ‘positive AI perception’ (65.7%) ^b^Summarized as ‘negative AI perception’ (13.7%)

The qualitative data confirm an optimistic view of AI’s potential, recognizing its ability to support and transform nursing. As visualized by the quotes, respondents expressed that AI can be a valuable relief in nursing, aiding everyday activities, optimizing work processes, and supporting data collection and interpretation, such as analyzing feelings or vital parameters. The ability of AI to enhance human-computer interaction is also recognized, particularly through AI-based voice control capabilities.

*’In the area of AI, we are at the beginning of the changes in nursing.’* (Text 4, para. 171)

*’AI has been part of our everyday lives for a long time. Whether as Siri [or] Alexa, AI tries to make everyday things easier or improve them. However, these are only minor peripheral areas, AI can recognize feelings and record vital parameters.’* (Text 1, para. 42)

*’AI means to optimize and support work and relieve employees.’* (Text 1, para. 74)

Moreover, nurses appreciate the role of AI in knowledge acquisition, including AI to answer questions, provide guidance, and aid in documentation. AI’s predictive capabilities are beneficial, allowing for early detection of care needs and risks, such as sepsis from care and laboratory parameters (Text 4, para. 170, Text 3, para. 152).

### Threats and negative AI perception

While AI presents numerous opportunities, such as simplifying tasks and making life easier, nurses recognize potential threats and risks. Negative perceptions are grounded in practical concerns, such as the risk of AI malfunction leading to mistakes. Some nurses fear that AI could replace human tasks. This is coupled with the understanding that nursing is fundamentally grounded in social relationships between nurses and patients, a dimension that AI might render impersonal. Other concerns include losing control over AI, which can cause feelings of helplessness and the high costs associated with AI implementation and maintenance (Text 4, para. 164).

*’I am afraid of AI stupidity, a system that delivers results after training that is not comprehensible because the sources are missing.’* (Text 1, para. 23)

*’AI threatens our jobs and makes care even more impersonal.’* (Text 1, para. 29)

*’It is also a question of refinancing. Unfortunately, it is often not possible for cost reasons to implement meaningful innovations or purchase them.’* (Text 4, para. 171)

### Profiteers and beneficial user groups

According to the quantitative findings, Table 7 illustrates the nurses’ perspectives on which outpatient and inpatient care groups could benefit most from AI. Nearly half of the nurses think that administration and management staff (49.5 %) can be supported most by AI, followed by nursing and support staff (25.3 %), patients in need of care (17.9 %), and social services and support (7.4 %).

**Table 7:** Most benefiting group of AI in nursing (self-intended, descending order, N=95)

Variable	Category	n ( %)
Which group of people benefits most from the use of AI?	Administration and management staff	47 (49.5 %)
	Nursing and support staff	24 (25.3 %)
	Patients in need of care	17 (17.9 %)
	Social services and support	7 (07.4 %)

In addition, nurses identified several parties who may profit from AI through qualitative data: (i) Micro Level - Physicians and hospital operators, (ii) Meso Level - Family nurses, social services, and service personnel, and (iii) Macro Level- Health and care insurers, government and policymakers, and nursing scientists. The advantages include more efficient processes such as invoice verification for insurers, early risk detection to contain healthcare costs, staffing savings for operators and employers, predictive care needs and assessment development in nursing science, and support for social participation in social services (Text 3, para 136 - 150).

### Needs for further AI implementation

Our qualitative data identifies potential facilitators, barriers, and requirements for successful AI implementation in nursing. The foremost necessity is the availability of reliable, structured, and complete data (Text 1, para. 55). Without such data, AI outcomes may be unsatisfactory (Text 1, Quote 41). The more accessible and trustworthy the data, the more robust the decisions the AI system makes. In addition to data considerations, human involvement is essential in shaping AI. This means active participation in its design and regulation, maintaining patient relationships, and fostering trust through sensitization and education about AI (Text 4, paras. 165, 166). Addressing fears that lead to feelings of helplessness, such as concerns over control and the possibility of rectifying unexpected outcomes, is also vital (Text 4, para. 164).

*’AI [...] must be fed with input to get a corresponding output.’* (Text 1, para. 11)

*’All groups of people will benefit only if they actively embrace this change. This is likely the greatest challenge, along with sensible refinancing.’* (Text 4, para. 165)

*’AI should be regulated, no one should lose her job through AI, and the relationship with patients must be preserved.’* (Text 4, para. 159)

*’Humans should be able to access and "reprogram" at any time.’* (Text 4, para. 164)

*’AI in route planning currently depends on too many parameters to work properly. AI should always be developed to provide maximum support to the nurse.’* (Text 4, para. 167)

As the quotes also conduct, user experience is another critical factor. AI solutions must be user-friendly, easy to use, and time-saving (Text 4, para. 161). Furthermore, the quotes show that AI solutions are still not satisfying enough, leading to the potential for improvements. Lastly, considerations regarding the costs and effort required for implementation should not be overlooked (Text 4, para. 165).

## Discussion

To answer the RQs, a cross-sectional online survey was conducted among nurses in Bavaria, Germany, in June 2023, utilizing a convenience sample. Our approach has provided comprehensive insights into nurses’ awareness and attitudes towards AI.

### Limitations

Our study provides valuable insights into nurses’ AI perception but is subject to several limitations. First, the concept of ‘perception’ itself is not clearly defined, potentially encompassing aspects of usage and acceptance, which visualizes the need for ground research. Our convenience sampling approach and the focus on Bavaria may not accurately reflect the broader nursing population, potentially introducing bias. The small sample size of 114 participants limits the study’s statistical power, particularly for conducting comprehensive multivariate analyses. We used bi-variate tests and calculated the chi-square. Still, nevertheless, the expected count in some cells was under 5 in 80 % of the cells, limiting our reliability and implicating the need for further research with bigger samples [36]. In summary, the small study sample constraint hinders our ability to explore interrelations and generalizations are limited.

Additionally, the study’s reliance on existing instruments like the BIDT study [22], while omitting broader models such as TAM or UTAUT may have affected the depth of theoretical exploration. The survey’s brevity could have resulted in an under-assessment of detailed nurse demographics and AI experiences. Furthermore, the inclusion and exclusion criteria are specific, focusing on formal nurses. Our study did not consider other healthcare professionals, missing out on comparative insights of AI perception across healthcare roles. Our cross-sectional design might not capture the evolving perceptions of AI, especially in the context of rapidly changing media narratives. Future research should consider more extensive and more varied sample sizes, incorporate comprehensive theoretical frameworks and adopt longitudinal methods.

### AI knowledge and attitudes

In addressing **RQ 1** on AI knowledge and attitudes in nursing, our study found that only 25.2 % of nurses have substantial AI knowledge, with the majority (73.0 %) having limited understanding. This aligns with existing research indicating a general lack of AI familiarity in healthcare [17, 19]. The need for increased AI awareness and training is evident [1, 20]. Furthermore, we confirmed the non-encompassing AI definition by nurses [7]. Nurses often perceive AI technically, which might contribute to skepticism and hinder acceptance. Our study also shows that some participants equate AI with humans, leading to a need to sensitize further for AI.

Although many nursing staff know too little about AI, two-thirds view AI optimistically, suggesting openness to AI implementation despite concerns about patient relationships, errors, costs, and job security. This is surprising and needs further research, as a lack of knowledge can be one of the constant causes of negative perception and low acceptance [34]. Furthermore, our open-ended data calls for functional, safe AI applications and emphasizes the importance of clear communication and nurses’ involvement in AI development to foster understanding and alleviate. This is also discussed in current literature to be important [1, 17, 19, 20]. In addition, studies about change management in nursing observed a higher success rate and acceptance of new technologies if nurses are involved and are part of the decision-making [38].

### AI application areas

For **RQ 2** the most known AI applications are (i) patient monitoring (55.7 %), (ii) route planning (47.7 %), and (iii) nursing documentation (43.2 %). Our qualitative data confirm these potentials. Nevertheless, open-ended data and the lack of knowledge let us assume that the use of AI in nursing care in Bavaria has so far made little inroads and that nurses have, therefore, hardly been confronted with AI in the working context.

Time studies in nursing highlight the considerable time spent on routine and administrative tasks, thereby supporting the use of AI in areas like monitoring and documentation [39, 40]. Current research, including works like Cho et al. [41], focuses on AI solutions for patient monitoring and clinical decision support. Our survey findings align with national literature [23], indicating that nurses are familiar with AI applications in administrative tasks, a trend expected to grow in the coming decade. Utilizing AI to streamline these processes can reduce bureaucratic workload.

### AI outcome and benefits

Addressing **RQ 3** on the expected outcomes and benefits of AI, our study indicates mostly positive perceptions. Nurses anticipate AI to alleviate the increasing burdens in nursing, particularly amidst current staff shortages and escalating workloads [9, 10]. As perceived by our respondents, the primary beneficiaries are administration and management (49.5%) and nursing staff (25.3%). This finding underscores the evolving roles in AI-integrated healthcare and aligns with Swan et al. [26].

Our results suggest starting AI implementation in administrative areas, supporting the recommendations of Chew et al.  [27], and extending to direct care. However, the apprehensions about AI expressed in some open-ended responses highlight the need for increased awareness and education across all potential user groups to mitigate concerns and foster a comprehensive understanding of AI’s role in nursing.

### Implications for nurses, technicians and policymakers

Our study’s findings lead to several critical implications and recommendations for nursing practice, policymakers, and healthcare facility management:

(i) Educational Campaigns: Increase public discourse and offer specialized training and further education for nursing staff to enhance their understanding of AI.
(ii) Reimbursement and Legal Frameworks: Set up clear reimbursement policies and legal frameworks that support the use of AI in nursing.
(iii) Investing in Nursing AI: Allocate resources for developing and implementing AI in nursing, ensuring user-friendliness.
(iv) Involvement of Nurses in AI Development: Engage nurses in the AI development.
(v) Provision of Technical Support: Deploy additional technical support and programming staff to assist nurses using AI tools in healthcare facilities.
(vi) Academization of Nursing: Integrate AI education into nursing curricula to prepare future nurses for the technological advancements in healthcare.

We emphasize that policymakers should prioritize the development and implementation of (i) targeted educational campaigns, (ii) reimbursement and legal frameworks that support the integration of AI in nursing, and (iii) investment into the development and implementation of nursing AI tailored to nursing professionals. Investments should include funding for AI research, establishing clear guidelines for AI application in healthcare, and ensuring ethical considerations are central to AI deployment in nursing settings. These straightforward guidelines should help to eliminate mistrust and negative perceptions, like seeing insecurities and threats to patient care as visualized rarely by our open-ended data. Investment and developments should focus on AI for patient monitoring (including patient allocation and fall detection), route planning, and nursing documentation, as nurses know them already and perceive them as promising areas. Administration and nursing management staff can be the most beneficial stakeholders, AI developers should ask themselves how to support nurses.

Moreover, technicians should (iv) involve nurses in developing AI applications through, e.g., focus groups. We recommend (v) that nurses get technical support in using AI tools effectively and aligning them with their needs. Nurses’ curricula should (vi) integrate AI education and prepare nurses for technological advancements.

## Conclusion

### Nurses AI perception takeaways

In summary, 74.8 % of our surveyed nurses lack a solid AI understanding. 65.7 % perceive AI positive as an opportunity. Our primary conclusion regarding the usage of AI is that nurses know patient monitoring, route planning, and nursing documentation as application fields. With the mounting pressure on nursing, AI is poised to play a vital role in alleviating healthcare staff and meeting the escalating nursing demands in Western Europe [10, 23]. Beneficial user groups are mostly administration and management staff (49.5 %) as well as nurses and their support staff (25.3 %).

Although our study is more indicative than conclusive, the finding that only 25.2% of respondents view themselves as AI experts highlights the need for increased AI education and sensitization in nursing. Mixed reactions, including positive views alongside rare concerns about job security and patient care changes, further underscore the importance of nurse involvement and education in AI development.

We anticipate a rise in AI knowledge and acceptance in nursing over the next decade, suggesting a promising future for AI integration. Our results indicate that administrative and routine tasks are prime areas for AI, making them strategic starting points for maximizing AI’s potential. Additionally, open-ended responses reveal a need for regulation, accessibility, and a focus on alleviating nurses’ workloads. We emphasize the importance of developing user-friendly AI that saves time on repetitive and administrative tasks, allowing nurses to focus on direct patient care.

### Further research

The limitations of our study and the national research gap necessitate further in-depth investigations into nurses’ comprehension, apprehensions, and expectations of AI. Subsequent research should build on our findings to deepen the understanding of AI’s role in healthcare. Essential areas for future research include applying new technologies in realistic settings and integrating AI knowledge into nursing education, ensuring its relevance and sustainability [42]. Studies should focus on assimilating AI knowledge into nursing training, especially considering nurses’ varying levels of familiarity. Nurses should be enabled to use new technologies, and the solutions should note real-world needs and should be integrated without a low threshold into practice, like our project ‘Smart Forest - 5G Clinics’ implies.

Further analysis should be with more extensive study samples and longitudinal to encompass broader data and compare AI perceptions across healthcare professions. Multivariate analysis should be conducted with new data, answering the question of what are influential factors to a good AI understanding and positive attitude towards AI. Besides, analyzing potential differences in perceptions, e.g., between nurses and doctors, is vital. Furthermore, exploring the real-world implementation of AI and its user acceptance are crucial, especially in light of our findings indicating nurses’ readiness to embrace AI as an opportunity rather than a risk.

### Supplementary Information

**Additional file 1.** Supplementary Material is available in the Appendix. The data set is available from both publication authors on request.

## Data Availability

The data sets generated during the study are not publicly available for data protection reasons but can be provided upon reasonable request from the authors.

## Footnotes

1 Simplifying the term AI acknowledges its complexity and aids in creating a practical research framework, bridging theory and practice in exploring AI’s applications.
