# Supplementary material for: Nurses’ perceptions, experience and knowledge regarding artificial intelligence: results from a cross-sectional online survey in Germany
Source: BMC Nurs. 2024 Mar 27;23:205. doi: 10.1186/s12912-024-01884-2 (PMC10967047; doi:10.1186/s12912-024-01884-2)
Supplement: Supplementary file 1 — Additional file 1. Supplementary Material is available in the Appendix. The data set is available from both publication authors on request. [file 12912_2024_1884_MOESM1_ESM.pdf]

Appendix A Questionaire

In the following appendix, the questions of our online survey are listed to visualize the content and to give other authors a chance to use our tool.

A.1 Startpage

Use of artificial intelligence (AI) in Care - *Survey by the Deggendorf Institute of Technology on the use of artificial intelligence (AI) in care.*

A warm welcome! Your opinion about using artificial intelligence (AI) in care is important to us! Your data is collected in a data protection-friendly manner and under high-security standards! **Here is the essential information on the survey:**

- The aim is to get a picture of the perception of AI.
- The information is to serve as a basis for the preparation of a publication and the derivation of improvement measures.
- The Deggendorf Institute of Technology surveys as part of the dissertation seminar of Lukas Schmidbauer and Domenic Sommer.
- It takes a maximum of 5 minutes to answer the questions. Your answers will be collected in a privacy-friendly way with your consent.

With your participation, you contribute to an important topic! Thank you!

A.2 Demographic data

1. How old are you?

   ☐ Under 20 ☐ 31-40 ☐ 51-60
   ☐ 20-30 ☐ 41-50 ☐ Over 60

2. Gender:

   ☐ Male ☐ Female ☐ Diverse

3. In what type of facility do you work?

   ☐ Inpatient long-term care ☐ Nursing (hospital/clinic)
   ☐ Outpatient care ☐ Other: ____________________

4. What is your highest level of education?
   ☐ No high school diploma
   ☐ Secondary school diploma
   ☐ Middle School (intermediate school leaving certificate) (German: Realschule)
   ☐ Higher School or specialized secondary school (German: Fach-/Abitur)
   ☐ Completed vocational training
   ☐ University degree (at least Bachelor's degree)
   ☐ Others: ____________________

A.3 AI knowledge (all self-assessment)

1. How much do you know about artificial intelligence (AI)?
   ☐ I would describe myself as an AI expert.
   ☐ I can explain well what is meant by it.
   ☐ I know roughly what is meant by it.
   ☐ I know the term, but I don't know what it means.
   ☐ I have not heard the term before.
2. Please describe in your own words what you understand by AI ... (maximum 1 sentence or character limit in LimeSurvey)
   ____
3. Which application areas of AI in nursing do you know? Please select all applicable options (multiple options in LimeSurvey). Please leave this question blank if you are unaware of any application areas.
   ☐ Nursing documentation
   ☐ Making nursing diagnoses
   ☐ Route planning
   ☐ Patient monitoring (e.g. vital signs, sleep)
   ☐ Patient care prediction (e.g., fall detection)
   ☐ Wound management
   ☐ Other: ____________________

A.4 AI Perception and personal conclusion

1. Do you see artificial intelligence in nursing more as an opportunity or a threat?
   ☐ Exclusively as an opportunity
   ☐ Rather as an opportunity
   ☐ Rather as a danger
   ☐ Exclusively as a danger
   ☐ I do not know, I cannot judge
2. Which group of people benefits most from the use of AI? Please indicate your priorities (sort in descending order of benefit)
   ☐ Nursing and support staff
   ☐ Social services and support
   ☐ Administration and management staff
   ☐ Patients in need of care
   ☐ Others: ____________________
3. Do you have any comments or feedback on AI? (Free text):
   ____
